# Improving lineament mapping using RISAT-1 SAR data, Nagpur and surrounding area in central India

**DOI:** 10.1016/j.dib.2023.109939

**Published:** 2023-12-12

**Authors:** Prabhakar K., Dhananjaya Rao E.N., Lakshmi Venkatesh A., Ankita Varma N., Aruna Ch.

**Affiliations:** Department of Geology, South Campus, Andhra University, Visakhapatnam, AP 530003, India

**Keywords:** LISS III, Microwave, Optical, HH Polarization, HV Polarization

## Abstract

Lineaments are the linear geological features can extend from few meters to hundreds of kms. Geologically lineaments are either structural or stratigraphical, typically it will comprise fault, fold axis, bedding contacts, dyke intrusions, shear zone or a straight coast line. Mapping lineaments using remote sensing is economical, faster can act as a preliminary study for geophysical survey such as gravity, electrical, magnetic and seismic etc. Generally, lineaments have been mapped using the optical remote sensing data such as Landsat, Resourcesat etc. For India, Lineaments were mapped using the LISS III and LISS IV of Resourcesat-1 & 2 at a scale of 1:50k. However in tropical region like India, limited exposure of ground due to vegetation cover, lineaments may go unnoticed in optical remote sensing data. This problem can be overcome by Synthetic Aperture Radar (SAR) data, which can penetrate ground significantly. With the launch of RISAT-1 satellite, data availability of SAR data is immense for Indian region. Aim of this study to explore the SAR data and merged SAR and optical data for lineament mapping.

Specifications Table

**Table:** 

Subject	Geology
Specific subject area	*Geology, Geographical Information System, Geotechnical Engineering and Engineering Geology*
Data format	Processed and Analysed RISAT remote sensing Raster data(.img) and shape files(.shp).
Type of data	SAR data of RISAT-1, LISS III optical Remote sensing data. Shape files. Table. Figure.
Data collection	Open-Source Data. All the data collected was freely available open-source data, which can be downloaded with login credentials. RISAT 1-SAR data was downloaded from the Bhoonidhi NRSC website. LISS-III optical remote sensing data from the NRSC Bhuvan and DEM data from USGS earth explorer website. ERDAS 2015 software was used to process the SAR and optical data. Arc GIS 10.8.2 software is for data generation, Lineaments digitization, and statistical calculations. Finally, Rockworks 17 software was utilized for creating the Rose Diagram plot. RISAT-1: https://bhoonidhi.nrsc.gov.in/bhoonidhi/index.html LISS II: https://bhuvan-app3.nrsc.gov.in/data/download/index.php?c=s&s=L3 DEM data : https://earthexplorer.usgs.gov ERDAS imagine : https://en.freedownloadmanager.org/users-choice/Erdas_Imagine_2015.html ArcGIS 10.8.2: https://desktop.arcgis.com/en/arcmap/latest/get-started/installation-guide/installing-on-your-computer.htm Rockworks 17 : https://www.rockware.com/support/rockworks-support/rockworks-downloads/rockworks-archived-versions/
Data source location	*19.6° to 22.2°N and 77.2° to 79.9°E, Nagpur and surrounding area in Central India.*
Data accessibility	Repository name: [Mendeley data] Data identification number: 10.17632/rs86jtwfn9.3 Direct URL to data: https://data.mendeley.com/datasets/rs86jtwfn9/3 kallempudi, prabhakar (2023), “Merged SAR and Optical”, Mendeley Data, V3, doi: 10.17632/rs86jtwfn9.3

## 1 Value of the Data

• This data can serve as a baseline for the delineation of lineament structures in inaccessible forest cover areas and play a crucial role in tectonics and engineering studies, regardless of weather conditions.
• The presented data can be used to implement lineament mapping structures in inaccessible areas and provide valuable insights into the underlying geology, geological processes and potential hazards.
• It is also useful for researchers and geomorphologists to identify topographic features and assist in activities such as landslide mapping and other engineering geology activities.
• It aids in understanding the geological, geomorphological, and environmental disturbances under study.

## 2 Background

Remote sensing has become the preliminary study on lineament identification on a regional scale. Optical Remote sensing data like Landsat, has extensively researched and applied in mapping lineaments. The lineaments extraction through remote sensing is well-established and has been the subject of numerous studies using optical data from sources such as Landsat, IRS, and SPOT., etc [1], [2], [3]. In tropical regions where vegetation covers the ground, it can be difficult to identify lineaments for mapping purposes [4]. Using SAR data, it is possible to study the sub-surface information while optical sensors record the surface. When radar and optical data are combined, they produce higher-quality images that can accurately interpret and map the details of structural attributes. A study by Rahman et al. in 2010 [5] utilized this merging technique. Similarly, Pal et al. in 2007 [6] used the merging of SAR and optical to identify geological and sub-surface structural features. Many works have compared and integrated images from optical and radar sources like ERS-1, ERS-2, and JERS-1 to interpret morpho-structural lineaments [7], [8], [9], [10].

## 3 Data Description

**Fig. 1:**
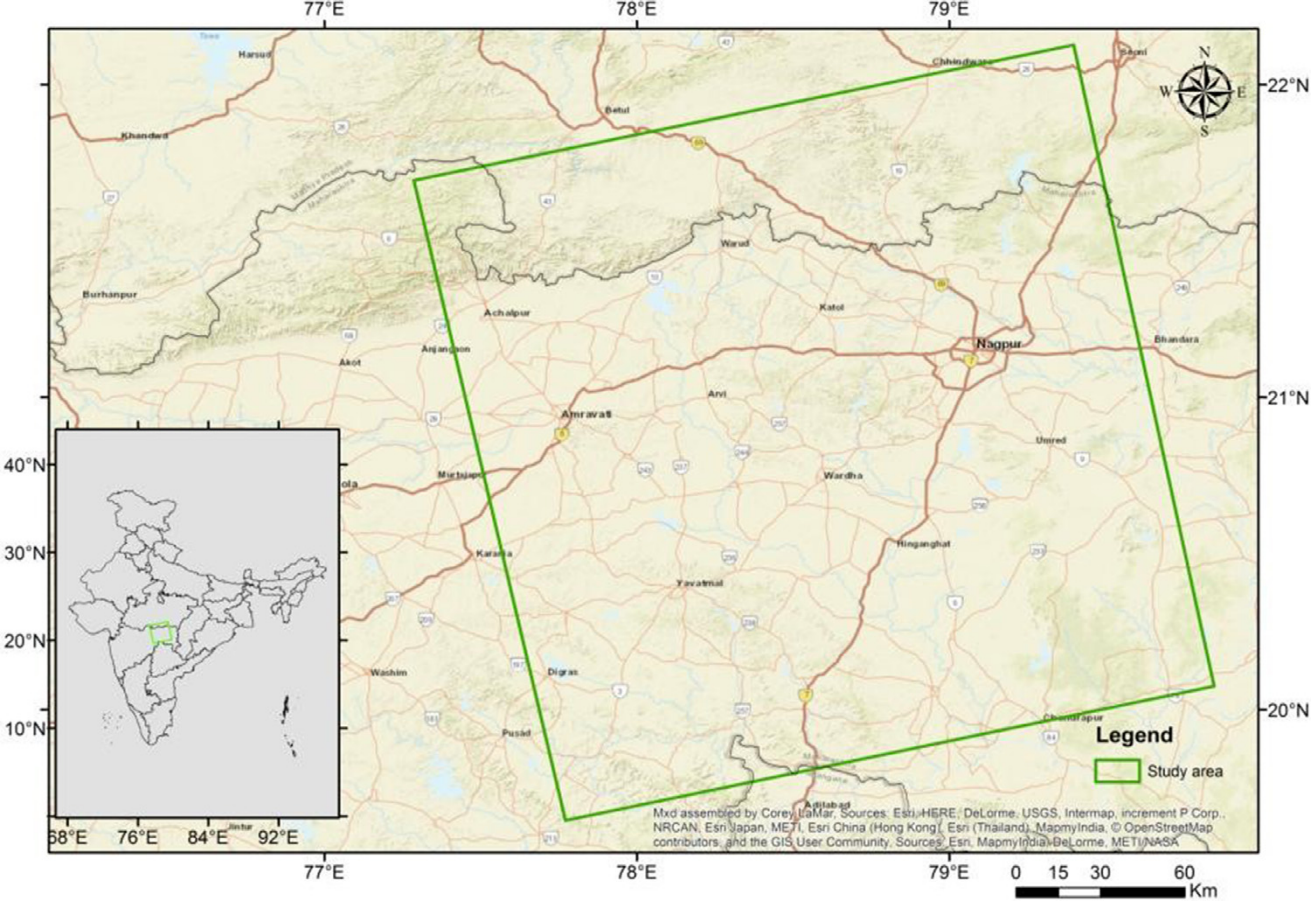
Study area.

**Fig. 2:**
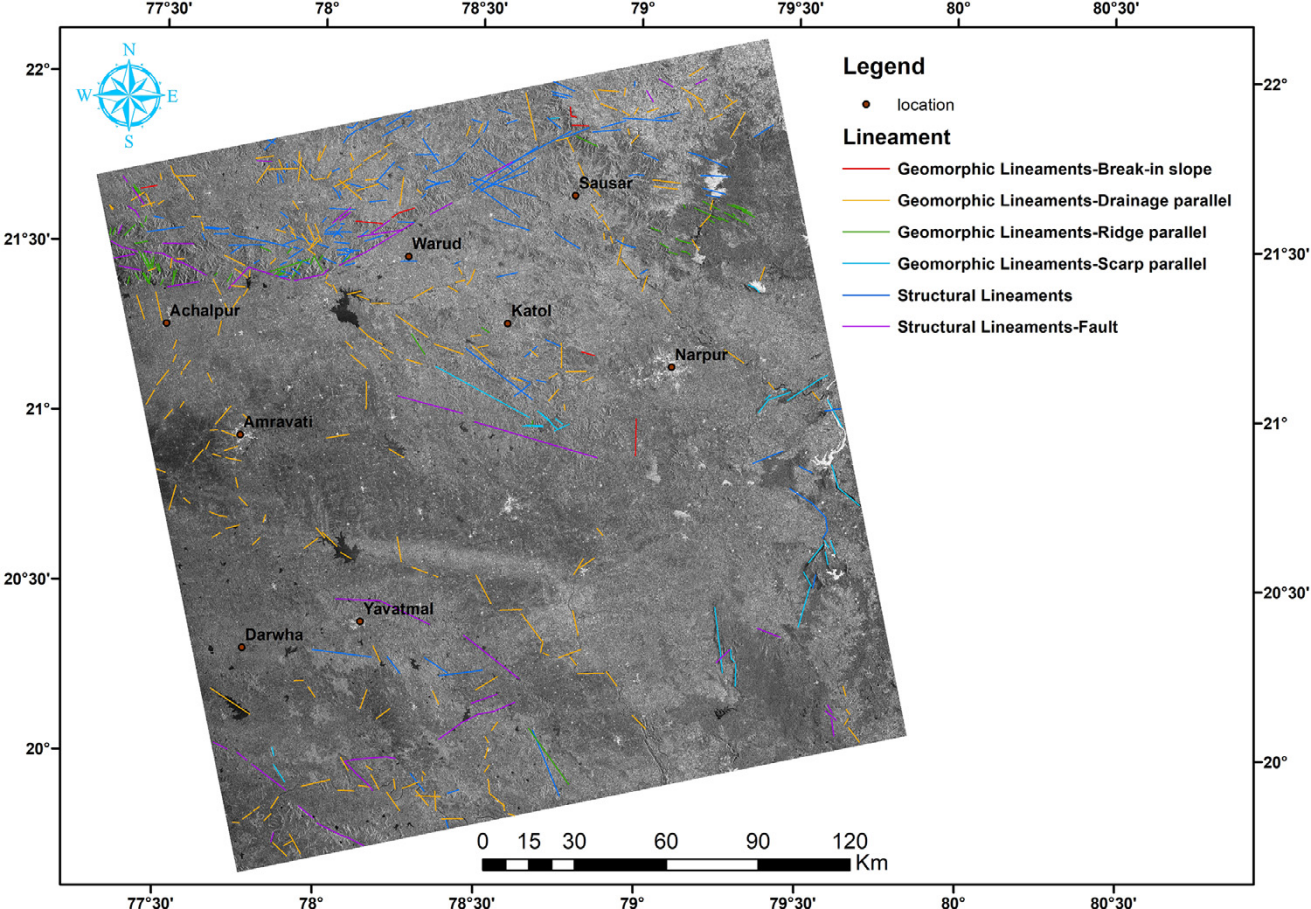
Lineaments in HH SAR data.

**Fig. 3:**
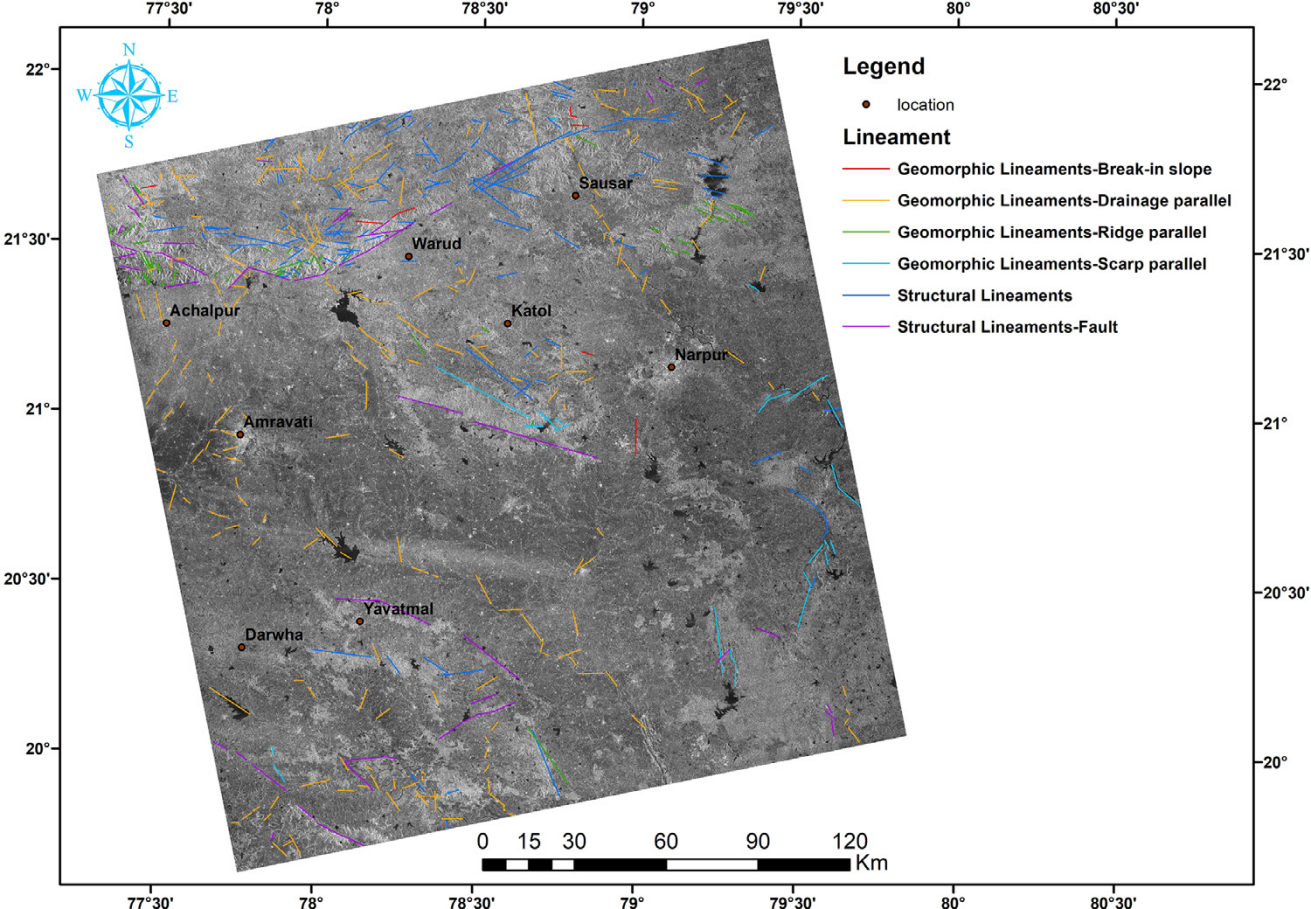
Lineaments in HV SAR data.

**Fig. 4:**
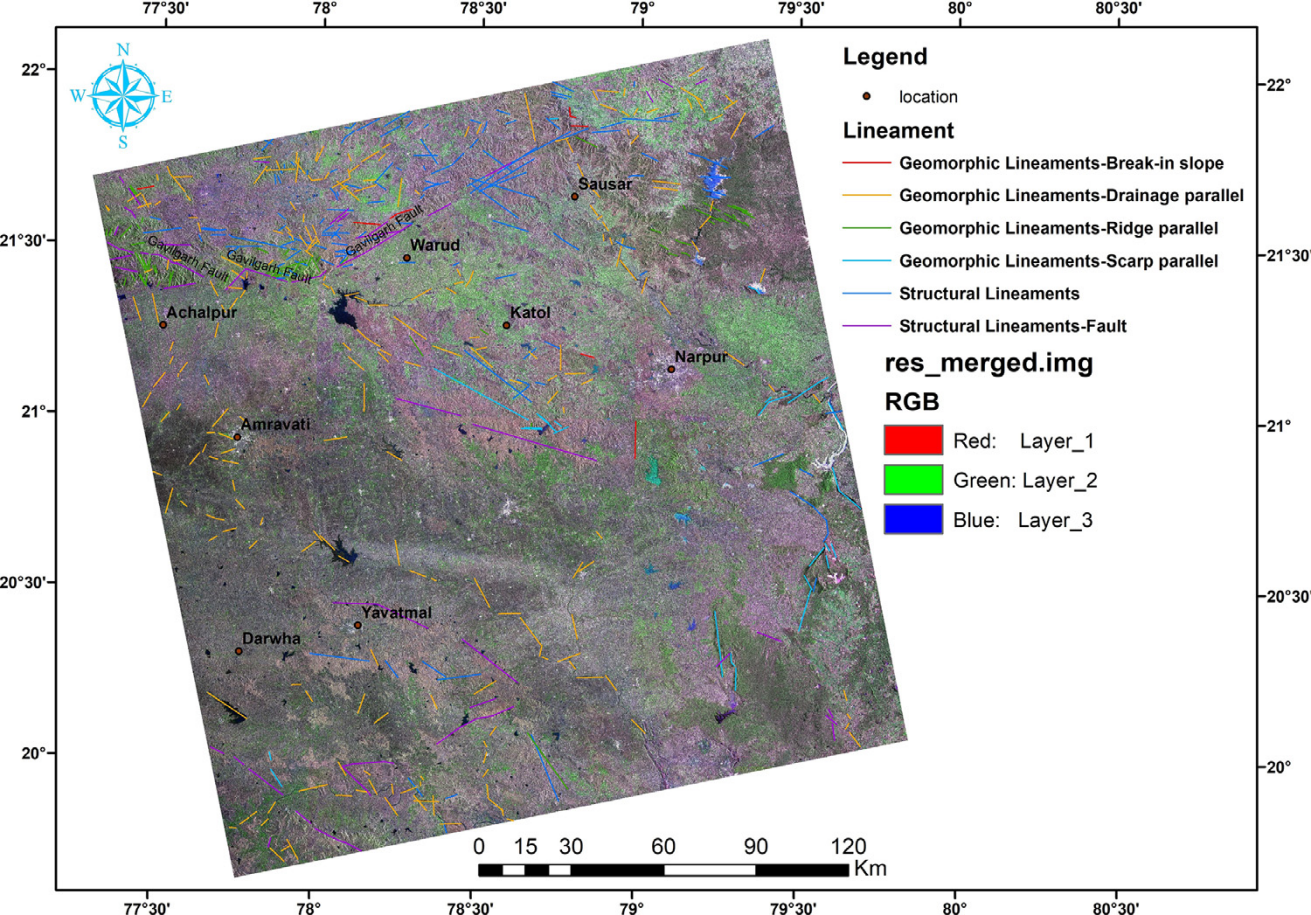
Merged optical and SAR data along with mapped lineaments.

**Fig. 5:**
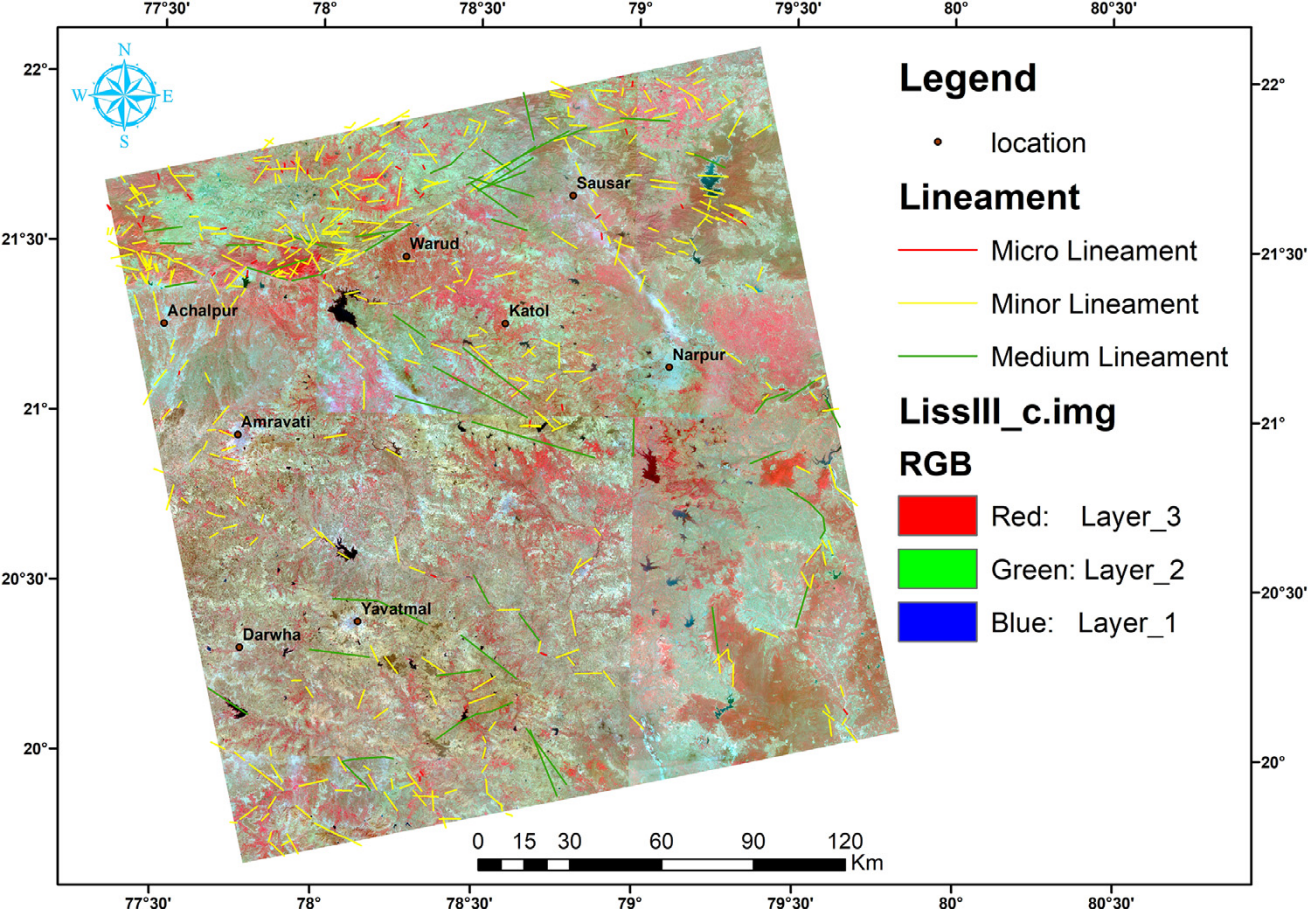
Lineaments in Optical data.

**Fig. 6:**
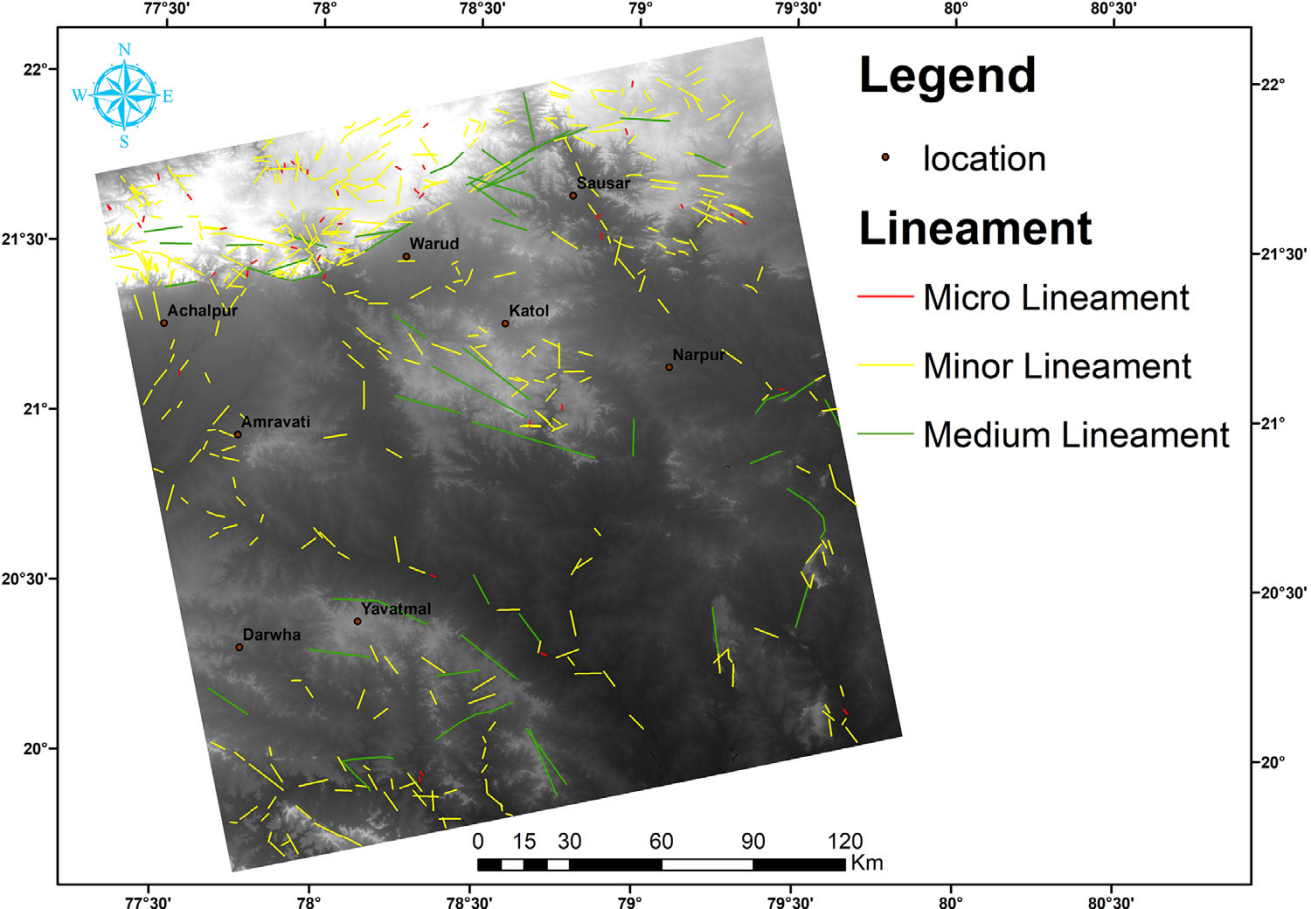
Lineaments in DEM data.

**Fig. 7:**
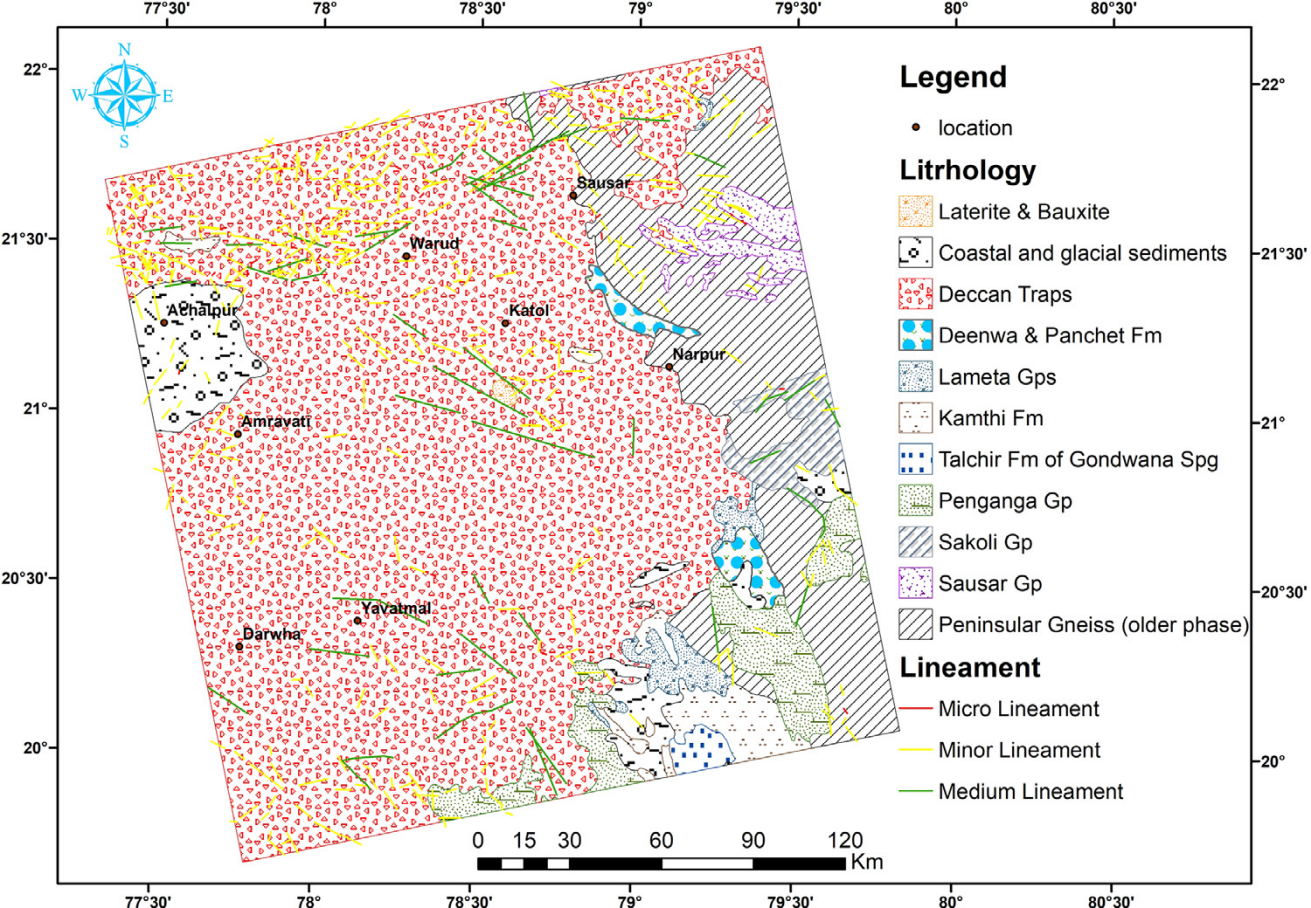
Lineament in Geology map.

In this article, we provide a Location map study area (Fig. 1), Lineaments in HH SAR data (Fig. 2), and HV SAR data (Fig. 3), Merged optical and SAR data (Fig. 4), LISS III Optical (Fig. 5), DEM (Fig. 6), Geology (Fig. 7) along with mapped lineaments.

### 3.1 Lab observation

A lineament is a linear feature on a surface that differs from adjacent features and may reflect a subsurface phenomenon. It can be simple or composite, aligning in either a rectilinear or slightly curvilinear relationship, Structural weaknesses in the ground can be identified through various features. These features can be a result of either natural tectonic disturbances or human activity.

These Lineament studies are helpful for economic, structural, and hazard assessment by predicting seismological and tectonic movements that give an in-depth understanding of the geological, geomorphological, and environmental disturbance.

Lineament mapping helps in knowing the structural and tectonic framework of an area and help plan mitigating efforts of any hazards arising from their response to neotectonic activity because these are pressure release zone to avoid large structures like dams and reservoirs. Lineaments are considered structural responses to lithospheric emplacement or displacement, cratonic activity, seismicity, orogenesis, plutonic activity, and metallogenesis.

Lineaments are classified based on their length, genesis, and geometry, extending from local to regional scales. Based on length, Mega >500 km, Major∼100-500 km, Medium∼ 10-100 km, Minor∼ 2-10 km, and Micro< 2 km. Mega lineaments are formed by a few significant features that play a crucial role in the tectonic evolution of a particular region. These lineaments can extend upto the basement rock and affect the entire area. They can be identified using low-resolution images, even with limited knowledge of the study area.

Major and medium lineaments are inferred as deep crustal fractures of magmatic episodes, acting as boundaries of basin or fault, and have a significant role in the tectonic evolution of that area and confined within the basin or rock of the same age. These are the linear features on the Earth's surface that represent various geological structures, including lithological contacts, axial plane fractures of folds, faults, and major fractures. Minor and Micro lineaments are the extensions/branches of the Major lineaments and contribute to the localization of mineralization, channelizing the groundwater or magma passage.

Traditionally, lineaments were identified with the help of contour maps and fieldwork investigations of geophysical surveys like gravity, electrical, magnetic, and seismic surveys, etc., which were time and money consuming methods and primarily depended on the extension of lineaments and accessibility of the area under investigation.

Remote sensing has the advantage of providing synoptic overviews on a regional scale and providing an easy way to identify lineament identification on a regional scale. Lineaments are the lines of contrast on satellite images. Most lineaments are easily identified based on hydrological properties because they have good moisture and vegetation content and may form specific drainage patterns easily detected on satellite images. More particularly, sudden changes in stream patterns, straight path of stream and/or vegetation, sudden disappearance/appearance of streams and/or springs in a line and sudden disappearance of vegetation or hill peaks, straight coastline features, and repetition of lithological parameters are the indications for identification of lineaments. Parallel drainage pattern with steep slopes indicates the presence of major faults, rectangular drainage pattern indicates the joints with a right angle to each other, and angular drainage pattern indicate the presence of joints and fault with an acute angle, etc. Fault or fault line scarp designates a sudden change in topography, vegetation, or shadow presence.

The backscattering of RADAR image data is primarily influenced by system parameters of wavelength, polarization, incidence angle, and terrain parameters of dielectric constant, surface roughness, and vegetation [11]. RADAR wavelengths can range from centimeters to meters. Shorter wavelengths are affected by atmospheric conditions due to interaction with water vapor, while longer wavelengths can penetrate through clouds to reveal the properties of surface soil. With increased wavelength, the skin depth also increases, allowing for longer penetration distances. This type of wavelength is ideal for lineament mapping.

If the radar system contains two antennas, it can take any polarized image like HH, HV, VH, or VV, whereas a radar system with a single antenna receives either co-polarized (HH/VV) or cross-polarized images (HV/VH). This difference in polarisation also helps in terrain identification. Rough surfaces appear brighter because of uniform radar return irrespective of the incidence angle whereas in smooth surfaces, the return signal decreases with decreasing depression angle and no return at lower depression angles. Materials with higher dielectric constants can better depolarize the incident EMR, increasing the intensity of the received signal and a brighter tone. The identification of geological features can be aided through the use of this property which can detect variations in lithology, moisture content, and salinity. The active SAR sensor's side-looking aspect produces radar “shadows” that highlight micro-relief and enhance structural geology. L-band radar images can detect coarse textural variations in outcrop and surface roughness, including faults, folds, topographic breaks, bedding, depressions, lithologies, and intrusive contacts [12]. In 2000, Robinson et al. [13] utilized SAR to detect paleo-channels, while Abdelsalam et al. [14] used it for sub-surface structural feature mapping. In 2012 study by De Oliveira Andrades Filho et al. [15], In northeastern Brazil, morphostructural lineaments were identified by combining SAR with a Digital Elevation Model. SAR imagery has proven useful in the geological mapping of glaciated and vegetated terrain. It has also been employed in investigations of structural geology related to the search for mineral deposits and hydrocarbon traps, as well as in the study of geologic hazards [12,[16], [17], [18], [19]].

In this study Radar Satellite-1 (RISAT-1) is used for delineation of lineaments. Radar Satellite-1 (RISAT-1) is a state of the art microwave remote sensing satellite carrying a Synthetic Aperture Radar (SAR) Payload operating in C-band (5.35 GHz), which enables imaging of the surface features during both day and night under all weather conditions. RISAT-1 was successfully launched by Indian Space Research Organisation, using PSLV-C19 on 26 April 2012. The RISAT can provide quad polarisation data (HH, VV, HV, VH) as well as circular polarizations.

The RISAT-1 can be operated in different modes ranging from spatial resolution of 3m in fine resolution mode with 30Km swath to 50m resolution with 240m resolution in Coarse resolution model data. In the absence of emergency or user request, the default mode of collection will be MRS descending, left looking, with dual polarization with a repeat cycle of 25 days with spatial resolution of 18-25m. The data can be acquired in all weather conditions, in both ascending (Evening) and descending mode (Morning) and in both right look and left look which means user can get data for any part of India at every 14 days interval.

The research zone is situated within the coordinates of 19.6° to 22.2°N and 77.2° to 79.9°E, encompassing an area of 51736 square kilometers across three states: Maharastra, Madhya Pradesh, and Telangana state (as shown in Fig.1). Nagpur, a major city in Maharashtra state and one of the nine districts in the Vidarbha region, is well-connected to every location in the study area. The northern part of the region consists of an undulating plain, with low hill ranges that belong to the Satpura hills strip (as shown in Fig. 6).

The geological makeup of the region consists of various rock formations. The Peninsular Gneiss belongs to the Archean age and is covered by Paleoproterozoic age rocks such as the Sakoli Group and Sausar Group, as well as Neoproterozoic age Penganga Group rock. The Gondwana formations include Talcher and Kamthi groups, while the Deccan Supergroups consist of basaltic lava flows, some of which are dense, compact, amygdaloidal, and friable. The cities falling under this category are Arvi, Betul, Chandur, Dathpur, Digras, Katol, Nagpur, and Wardha. Recent formations of laterites and sediments cover these rock formations. The west of Nagpur is mainly occupied by the Deccan Trap formation, while the east is occupied by the metamorphic and crystalline series. The junction of these traps and crystalline formations is occupied by Lameta and the Gondwana formation (Fig. 7).

**Fig. 8:**
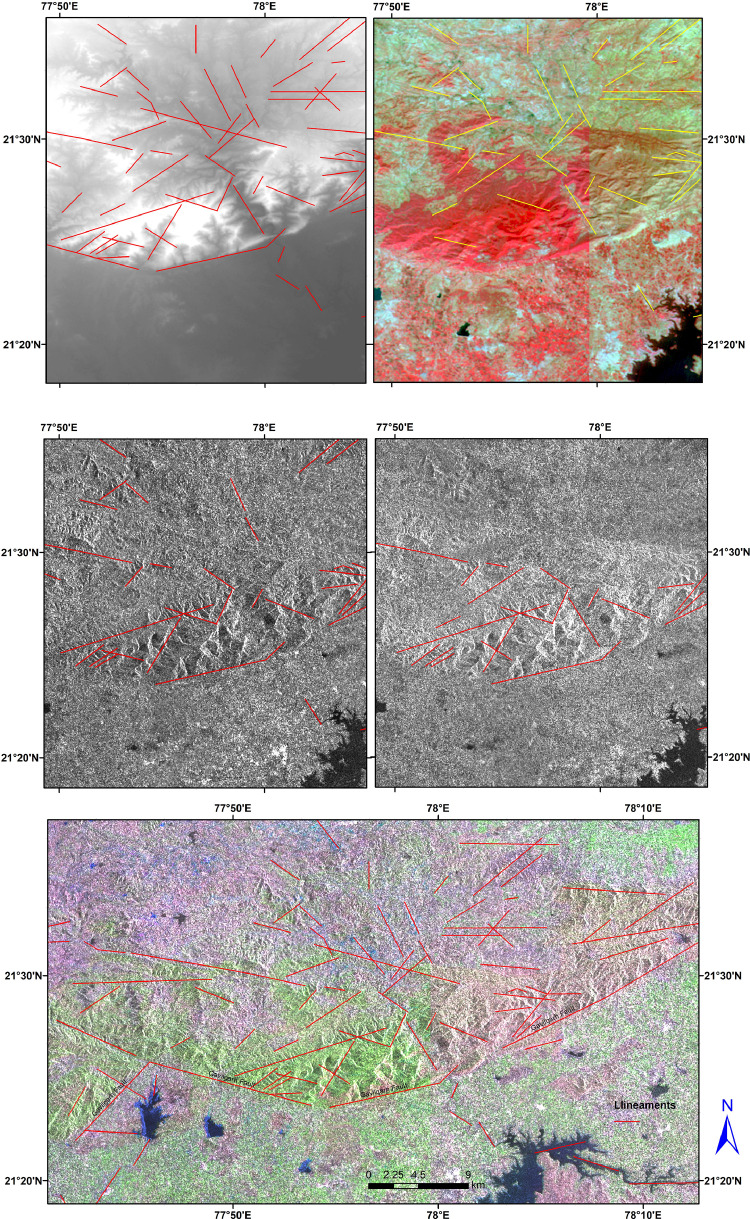
Comparison of lineament mapping in a) DEM b) Optical image c) HH SAR image d) HV SAR image e) SAR+Optical image.

The difference in HH and HV polarization images (Figs. 2 and 3) were used for the identification of the relief variations and the terrain variations interns which helped in the mapping of lineaments. The NW part of the area is known as Gavilgarh Fault Zone, Different polarisation images show variations in Scarp Heights ranging between 200 mt and 400 mt, which aid in the identification of lineaments (Fig. 8c,d and Comparison of lineament mapping.mpk file in repository). The degraded scarp face at the foothills, which is the geomorphic signature of Gavilgarh Fault Zone, is revealed in the merged SAR image (Fig. 8e). After merging Optical with SAR, Cuesta/ hogback structures, triangular facets, straight valleys, Alluvial fan of Quaternary sediments, and zones of river anomaly are visible, providing evidence of Quaternary tectonism in the study area. (Fig. 8d and Comparison of lineament mapping.mpk file in repository). These lineaments play a significant role in controlling the flow of rivers in the eastern and southern regions, as shown in Fig. 7.

Lineaments are conducive to vegetation growth and can be easily identified using optical data, as demonstrated in Fig. 5. To generate a comprehensive map of lineaments, both optical and SAR data were combined, as shown in Fig. 4. By examining tonal, textural and relief variations in the DEM and merged data, lineaments were effectively mapped, as shown in Fig. 6.

In this study area, 516 lineaments were identified through visual observation and key identification points. The data from Optical and SAR revealed an increase in the number of lineaments and their genetic classification (merged.mpk file in repository). According to the lengthwise classification, there are 44 (8.5271%) micro lineaments, 425 (82.364%%) minor lineaments, 47 (9.108%%) medium lineaments, and no major or mega lineaments present ( Fig. 6), which suggests the tectonic nature of the area. Most of these lineaments are structural lineaments such as faults and crushed zones, while few are considered geomorphologic lineaments. Rose diagram infer that most of the lineaments and structural lineaments oriented in E-W direction (Fig. 9a,Fig. 9c) where Geomorphic lineaments are symmetrical in all direction (Fig. 9b). Some lineaments were observed in the central part of the map based on tonal variations and river paths (The Wardha River and Maru River) and the line of ponds (Fig. 4).

**Fig. 9a:**
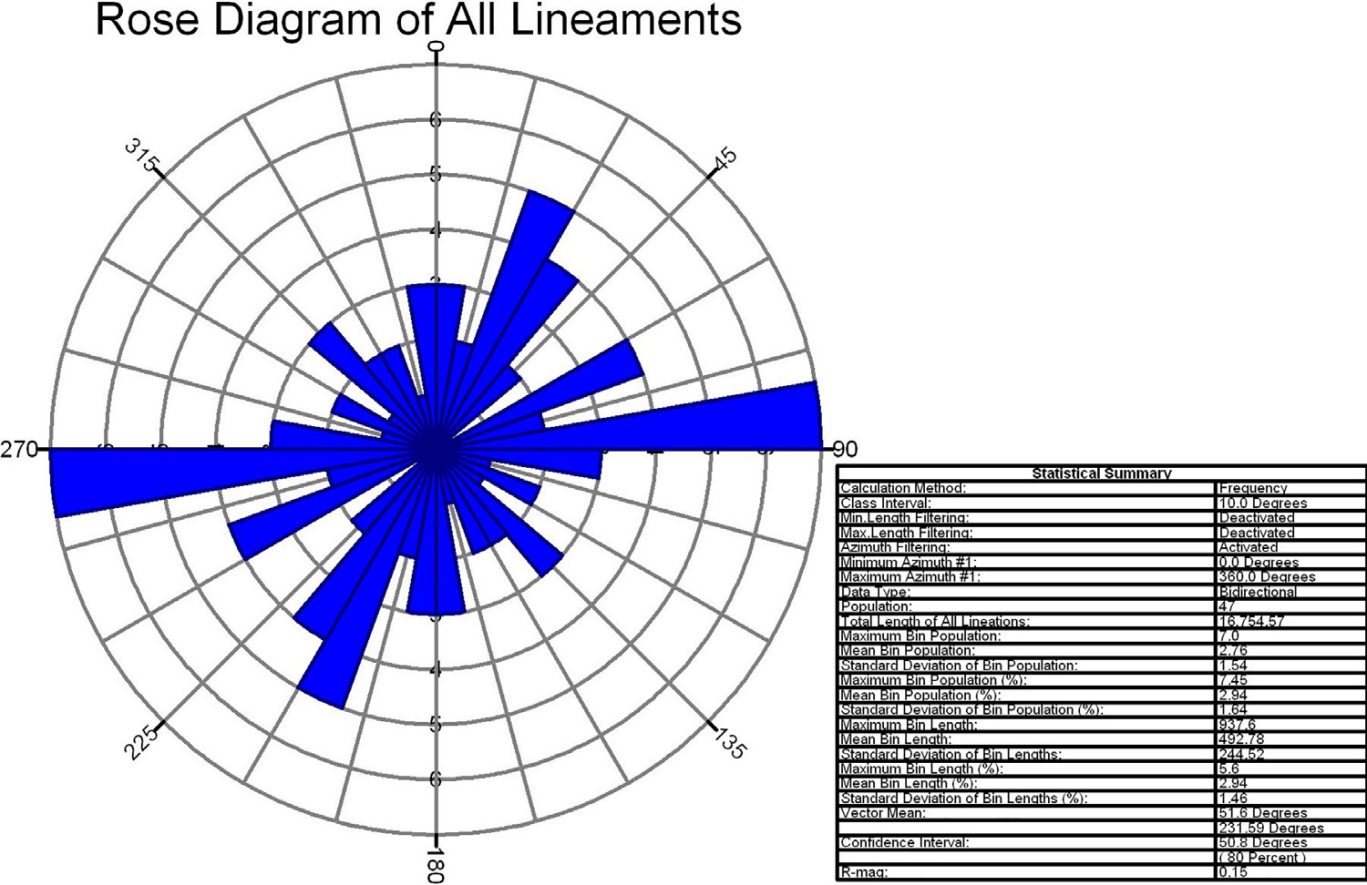
Rose Diagram of Azimuthal trend of all lineaments.

**Fig. 9b:**
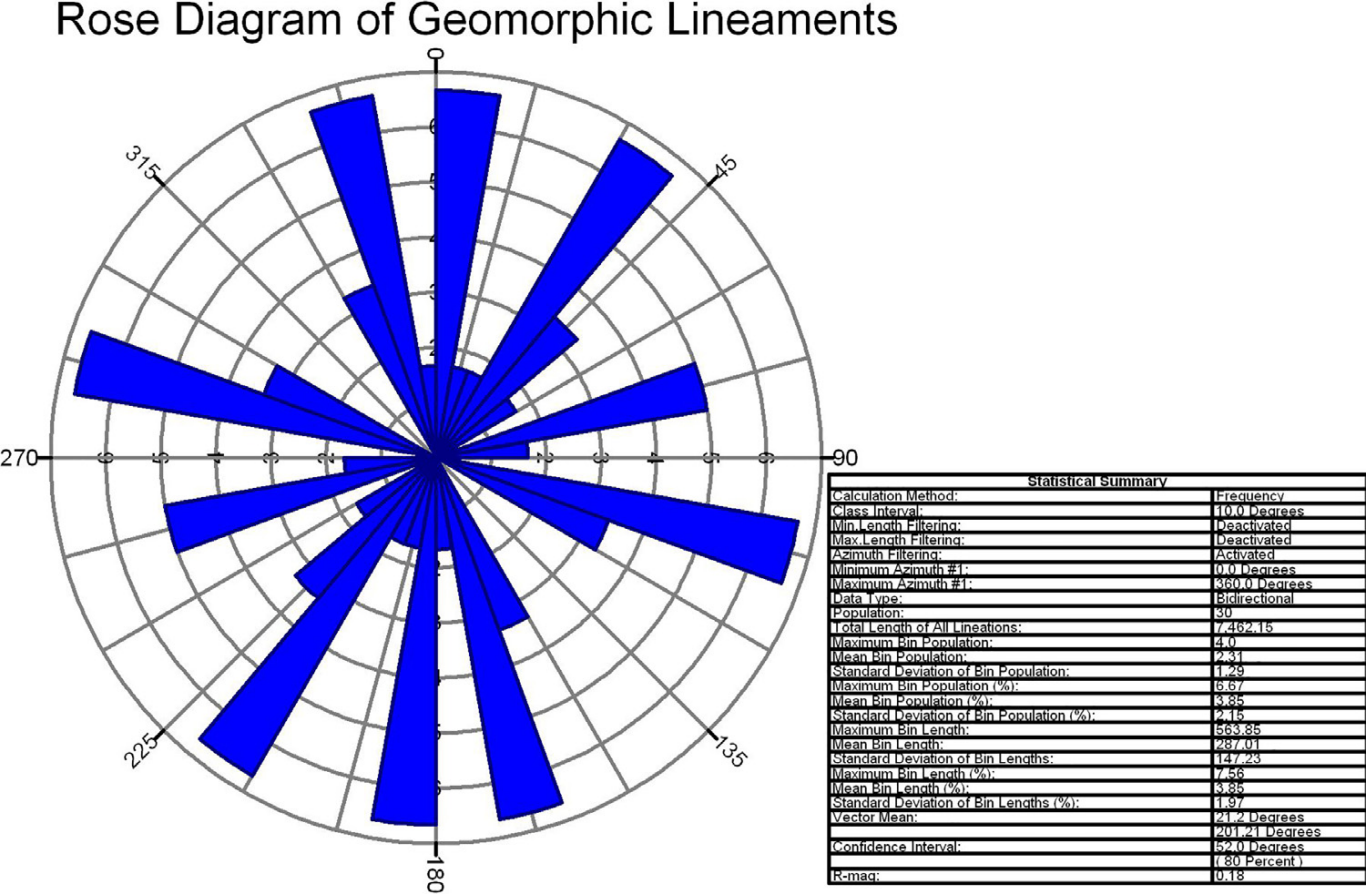
Rose Diagram of Azimuthal trend of geomorphic lineaments.

**Fig. 9c:**
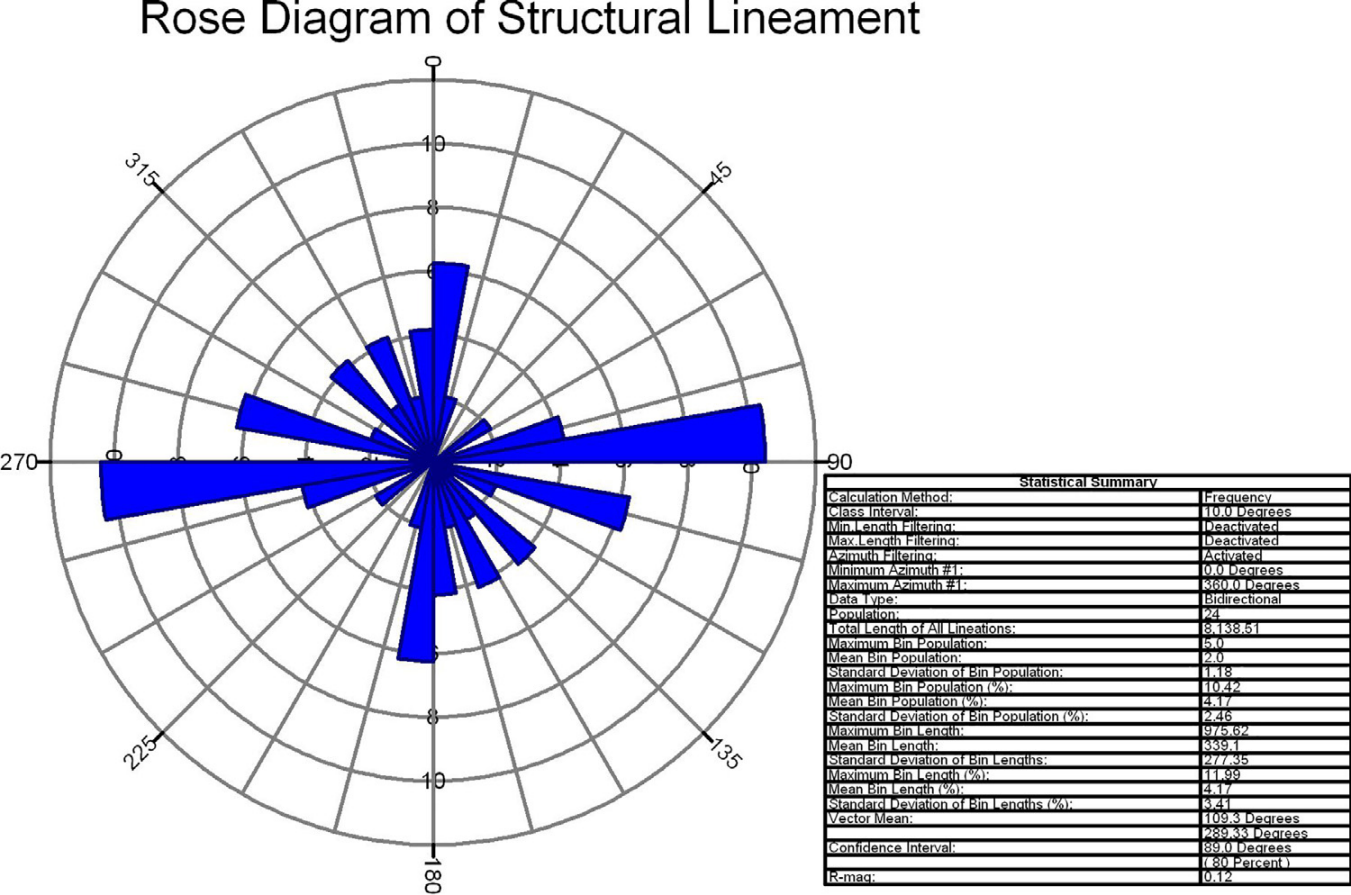
Rose Diagram of Azimuthal trend of structural lineaments.

## 4 Experimental Design, Materials and Methods

### 4.1 Data collection

Downloaded the RISAT 1 satellite data from the Bhoonidhi- ISRO, LISS III data from NRSC Bhuwan and DEM from USGS earth explorer website. Regional geology (1:50,000) was downloaded from GSI- Bhukosh website.

### 4.2 Processing data and methodology

The map package files (merged.mpk file in repository) were prepared using Arc GIS 10.8.2 and ERDAS 2015 softwares. The data files include the SAR data in HH, HV- polarizations, DEM, and IRS LISS III data with the 30m spatial resolution. In the present study dual polarisation (HH, HV) with the spatial resolution of 18m is used. In addition to SAR data, IRS LISS III data was used which contains four spectral bands ranging from Optical to Near Infra Red (NIR) with the 30m spatial resolution. Resolution merge of SAR and optical data were done using Brovey transform in ERDAS 2015 software. Keeping the display at 10%% and background values (R, G, B) at zero is used for better visualization and digitization of lineaments.

Lineaments digitization, statistical calculation, data generation, comparison of different data sets (merged.mpk file in repository) are prepared Using ArcGIS 10.8.2 software. With the help of the lineament shape file attribute table calculated all statistics. Split line tool and other geometric calculations were done in ArcGIS giving the Azimuthal trend of each lineament. Rose diagram has been prepared in frequency calculation method from the Azimuthal trend of each lineament (Rose diagram data.rar file in repository) with the help of Rockwork17 software. Importing the data into the linears tool in utilities of Rockworks17 software. All the data and software are open-access and freely available.

A study was conducted to investigate lineament mapping using both SAR and optical data. The results showed that incorporating SAR data into the mapping process improved accuracy, as it provided additional information on relief and structural characteristics that complemented the vegetation and stream-related data obtained through optical data. The mapping was carried out using individual HH, HV images, and resolution merging of SAR and optical data. The study recommends using RISAT-1 data in combination with Resourcesat-2 data for lineament mapping in India. While the study did not cover the use of DEM data, it was observed that including it could lead to the identification of more lineaments. Therefore, a combination of SAR (RISAT-1), optical (Resourcesat-2), and DEM (Cartosat-1) data is recommended for effective lineament mapping.

## Limitations

None.

## Ethics Statement

This article does not contain any studies with human or animal subjects and data did not collected from social media platforms.

## CRediT authorship contribution statement

**Prabhakar K.:** Conceptualization, Methodology, Software, Resources, Data curation, Writing – original draft, Writing – review & editing. **Dhananjaya Rao E.N.:** Supervision. **Lakshmi Venkatesh A.:** Resources, Investigation. **Ankita Varma N.:** Resources. **Aruna Ch.:** Resources.

## Data Availability

Merged SAR and Optical (Original data) (Mendeley Data)
